# Outcomes in critically ill patients with cancer-related complications

**DOI:** 10.1186/cc14685

**Published:** 2015-09-28

**Authors:** Viviane BL Torres, Juliana Vassalo, Nelson Spector, Fernando A Bozza, Jorge IF Salluh, Marcio Soares

**Affiliations:** 1 Postgraduate Program in Internal Medicine, School of Medicine, UFRJ, Rio de Janeiro, RJ, Brazil; 2 IDOR--D'Or Institute for Research and Education, Rio de Janeiro, RJ, Brazil; 3 Postgraduate Program, Instituto Nacional de Câncer, Rio de Janeiro, RE, Brazil

## Introduction

Cancer patients are at risk for severe complications related to the underlying malignancy or its treatment, and therefore usually require admission to ICUs.

## Objective

To evaluate the clinical characteristics and outcomes in this subgroup of patients.

## Methods

Analysis of two prospective cohorts of cancer patients admitted to ICUs. We used multivariable logistic regression to identify variables associated with hospital mortality.

## Results

Out of 2028 patients, 456 (23 %) had cancer-related complications. Compared with those without complications, they more frequently had worse performance status (PS) (57 % vs. 36 % with PS ≥2), active malignancy (43 % vs. 5 %), need for vasopressors (45 % vs. 34 %), mechanical ventilation (70 % vs. 51 %) and dialysis (12 % vs. 8 %) (*P *<0.001 for all analyses). ICU (47 % vs. 27 %) and hospital (63 % vs. 38 %) mortality rates were also higher in patients with complications (*P *<0.001). Chemo/radiation therapy-induced toxicity (6 %), venous thromboembolism (5 %), respiratory failure (4 %), gastrointestinal involvement (3 %) and vena cava syndrome (VCS) (2 %) were the most frequent complications. In multivariable analysis, the presence of cancer-related complications per se was not associated with mortality (odds ratio (OR) = 1.25 (95 % confidence interval, 0.94-1.66)). However, among the individual complications, VCS (OR = 3.79 (1.11-12.92)), gastrointestinal involvement (OR = 3.05 (1.57-5.91)) and respiratory failure (OR = 1.96 (1.04-3.71)) were independently associated with worse outcomes.

## Conclusion

The prognostic impact of cancer-related complications was variable. Although some complications were associated with worse outcomes, the presence of a severe acute cancer-related complication per se should not guide decisions to admit a patient to the ICU.

